# The impact of physical activity on cumulative cardiovascular disease risk factors among Malaysian adults

**DOI:** 10.1186/s12889-015-2577-5

**Published:** 2015-12-16

**Authors:** Rajah Rasiah, Govindamal Thangiah, Khalid Yusoff, Rishya Manikam, Sankara Kumar Chandrasekaran, Rujhan Mustafa, Najmin Binti Abu Bakar

**Affiliations:** Department of Development Studies, Faculty of Economics and Administration, University of Malaya, 50603 Kuala Lumpur, Malaysia; Faculty of Medicine, University of Malaya, 50603 Kuala Lumpur, Malaysia; Faculty of Medicine, University Teknologi MARA (UiTM), 47000 Sungai Buloh, Selangor Malaysia; Malaysian Qualifying Agency, Petaling Jaya, Malaysia

**Keywords:** Cumulative cardiovascular disease risk factors, Physical activity, Socio-demographic factors

## Abstract

**Background:**

Numerous studies have shown the importance of physical activity in reducing the morbidity and mortality rates caused by cardiovascular disease (CVD). However, most of these studies emphasise little on the cumulative effect of CVD risk factors. Hence, this study investigates the association between physical exercise and cumulative CVD risk factors among adults in three different age groups.

**Methods:**

Using a sample of 7276 respondents drawn from community centers, the REDISCOVER team gathered information on physical activity, CVD risk factors (obesity, diabetes, hypertension, hypercholesterolemia, tobacco use) and socioeconomic and demographic variables in Malaysia. Because the study required medical examination, a convenience sampling frame was preferred in which all volunteers were included in the study. Fasting blood samples and anthropometric (height, weight and more) measurements were collected by trained staffs. Socio-demographic and physical activity variables were recorded through questionnaires. A Chi-square test was performed to identify the bivariate association between the covariates (socioeconomic variables, demographic variables and physical activity) and outcome variable. The association between the main exposure, physical activity, and the outcome variable, cumulative CVD risk factors, was assessed using an ordinal logistic regression model, controlling for socioeconomic status and demographic influences in three different age groups, 35–49, 50–64 and 65 and above.

**Results:**

The mean age of participants is 51.8 (SD = 9.4). Respondents in the age groups of 35–49 (aOR_moderate_ = 0.12; 95 % CI: 0.02 - 0.53 ) and 65 and above (aOR_high_ = 0.58; 95 % CI: 0.24, 0.78) showed a statistically significant inverse relationship between physical activity and cumulative CVD risk factors. However, this relationship was not significant among respondents in the 50–64 age group suggesting the possible influence of other variables, such as stress and environment.

**Conclusions:**

The statistically significant results show a negative association between physical exercise and cumulative CVD risk factors. However, the lack of a significant relationship in the 50–64 age group suggests the need to include other considerations in future studies, such as stress and environment.

## Background

The life expectancy of adults, among other things depends on their lifestyle, which includes physical activity. Physical activity is a well-recognized modifiable risk factor of chronic diseases, such as breast cancer, colon cancer, cardiovascular diseases (CVD) [1–5]. Past studies have shown that running, walking, jogging and other forms of exercise helps protect individuals from ill health. Since the seminal work of Morris et al., the benefits of physical activity are well documented and acknowledged [6]. Among the advantages include low level of depression, stress, mortality and morbidity of chronic diseases and increasing strength of bones and muscles, which also prevents arthritis and joint pains [7].

Despite the increasing evidence on the positive effects of physical activity in keeping several diseases in check, many remain physically inactive. Thus, physical inactivity has become an alarming public health problem [8]. Physical inactivity alone has contributed to 5.3 million deaths annually around the globe. Furthermore, available data shows that 31.1 % of the world’s population did not meet any of the of three physical activity recommendations of 30 min of moderate-intensity physical activity on at least 5 days a week, 20 min of vigorous –intensity physical activity on at least 3 days every week or an equivalent combination achieving 600 metabolic equivalent (MET)- minute per week [9].

Another study showed that diabetes and coronary heart disease (CHD) in 7 % and 6 % of the world’s population respectively could be reduced if they achieved these physical activity recommendations [3]. Similarly, among countries in the Pacific region, Malaysia had the third and second highest rates of diabetes and CHD [3]. Increased physical inactivity is believed to be caused by rising urbanization and development processes arising from the introduction of technologies and machineries that promote labour-saving and sedentary lifestyles [10, 11] which causes high rates of mortality and morbidity.

Among the non-communicable diseases (NCD), CVD is the leading cause of death worldwide, which is particularly a serious economic burden among the middle and low income countries. CVD risk factors affects households, labour productivity and human capital development. At the household level, CVD thrusts people into a vicious cycle of poverty that often causes the liquidation of assets and immiserisation [12]. Therefore, it is important to identify factors that can lower the incidence of CVD risk factors. A study using data from University of Pennsylvania Alumni Health Study showed that those with high physical activity (> = 3500 kcal per week) had low incidence of diabetes (13.7 cases per 10,000 man-years of observation) compared to those with low physical activity [13]. Similarly, in Finland, women and men had low CVD related mortality rates due to increasing activity, occupational activity and commuting activity [14]. In contrast, in Chennai, India, people who performed light physical activities experienced high rates of diabetes, hypertension, obesity and abdominal obesity, which was highly significant [15].

Also, the EPIC-Norfolk study in United Kingdom showed that both male and female participants with metabolic syndrome show a fall in risk of developing CHD as the physical activity status changes from very inactive to very active (men: 21.8 % to11.5 %; women: 12.4 % to 5.3 %) [16]. Another evidence from Norway showed a decreasing trend in hypertension as physical activity increased from low (50 %) to high (38 %) for those in the age group of 45 to 64 (significant at 1 %) and more than 65 years old (significant at 10 %) [17]. A similar pattern was observed for diabetes and body mass index (BMI) among those above 65 years old and all age groups [17]. The Women’s Health Study showed that women with high physical activity (kilocalories per week) had better CVD risk profiles compared to physically inactive women [18].

However, the results of some studies differ from the above findings. For example, one study argued that exercise have triggered 54 cases of myocardial infarction among 1228 patients who are confirmed with acute myocardial infarction [19]. Another study from Europe showed increasing systolic and diastolic blood pressure with work related activity and household chores [20]. Similarly, in Spain, both men and women showed high levels of BMI and obesity with increasing work related physical activity, though it became insignificant once controlled for potential confounding variables [21].

In light of the contrasting findings, we aim in this paper to revisit the relationship between physical activity and CVD risk factors. We chose Malaysia for the study because it had the second and third highest incidence of diabetes and CHD respectively in the Pacific region. In Malaysia, more than half (61.4 %) of the people are insufficiently physically active with females being more physically inactive than men [9]. The Malaysian Adult Nutrition Survey (MANS) study in 2003 showed that 12.8 % of the population were physically active while 39.7 % and 47.6 % were sedentary and moderately active respectively [22] , and these shares are believed to be rising. Therefore, it is crucial to investigate the consequence of physical inactivity and its relationship with the prevalence of CVD risk factors in a middle income country. Also, most related studies on the developing countries had focused on a single CVD risk factor. Very few studies have investigated the effect of physical inactivity on cumulative CVD risk factors in the developing countries. Therefore, this study examines the impact of physical activity on cumulative CVD risk factors among adults in three different age groups, in Malaysia which is a middle income country.

## Methods

A sample of 7276 adults aged 35 and above participated in this community-based health survey by the REDISCOVER team following the same convenience sampling approach used by Yusuf et al.[23] that yielded a high participation rate. The convenience sampling design was conducted to enable a thorough health screening and also to ensure the feasibility for a long-term follow-up study. However, the present study is based on a baseline survey over the period from 2007 to 2010. Respondents were briefed that this was a survey conducted by trained enumerators and staffs. Although the sample is not broad as it includes only respondents who agreed to participate, it covers different geographical areas and states in Malaysia with substantial heterogeneity in socioeconomic status, culture and physical environment.

The recruited participants were gathered at their community centres located in Selangor, Federal territory of Kuala Lumpur, Negeri Sembilan, Pahang, Kelantan and Sabah. Only those who gave a written consent were enrolled in this study. A large number of participants agreed to be part of this study due to the pledge made by the REDISCOVER team to screen respondent’s CVD risk factors from 2007 to 2016 and also to treat those with CVD for free at public hospitals. The high density lipoprotein (HDL), low density lipoprotein (LDL), systolic and diastolic blood pressure levels, glucose levels, tobacco use, waist to hip circumference, and body mass index (BMI) are recorded from the participants. Their demographic (age, gender, location, ethnicity) and socioeconomic variables (income groups, employment status) were recorded through a questionnaire. The study was approved by the Ethics Committee of Universiti Teknologi Mara (UiTM) in 2007.

### Outcome

#### CVD risk factors

Obesity. Obesity refers to a body mass index (BMI) ≥ 27.5 kg/m^2^, which was measured by weight in kilograms divided by the square of height in meters. Abdominal obesity is defined as the waist to hip ratio based on the measurements taken for men > 0.90 and women > 0.80 [24].

Hypertension. Hypertension refer to high blood pressure which was measured in millimetres of mercury (mm/Hg) and occurs when the systolic and diastolic reading exceeds 140 mm/Hg and 90 mm/Hg respectively [25]. Also, participants who are diagnosed with hypertension and who are under high blood pressure medications were classified as hypertensive patients.

Hypercholesterolemia. Hypercholesterolemia was measured in millimoles per liter of blood (mmol/L). Hypercholesterolemia was detected for participants with total cholesterol (TC), high density lipoprotein (HDL) and low density lipoprotein (LDL) levels greater than 5.2 mmol/L [25], 1.6 mmol/L and 4.2 mmol/L respectively. Patients, who are under cholesterol medication, were also classified as high cholesterol patients.

Diabetes. A reading of fasting glucose level ≥ 7.0 mmol/L indicates the occurrence of diabetes [25]. Patients diagnosed with diabetes, as well as those under medication, such as oral hypoglycaemic and insulin injections were classified as diabetic.

Tobacco Use. Participants were categorized into three groups based on their history of tobacco use, which were, have used tobacco products in the past, currently using tobacco products and never used tobacco products.

Each of these risk factors were treated as dummy variables (0, 1) with 1 indicating the presence of the risk factor and 0 otherwise. All the risk factors were then summed to yield the cumulative CVD risk factors, which ranged from 0 to 5. They were then converted into four groups of cumulative CVD risk factors, namely, no risk factors, one risk factor, two risk factors and three or more risk factors. Three or more risk factors category was formed by including respondents that have 3, 4 or 5 risk factors. The sub-categories of 4 (560) and 5 (29) risk factors had few observations. Hence we integrated them into the fourth category to satisfy the relevant proportional odds assumption of the ordinal logistic regression applied in the study. The cumulative CVD risk factor was used as the dependent or outcome variable where CVD risk factors were weighted equally [26].

### Predictor variable

#### Physical activity assessment

Physical activity was assessed in four different domains: namely, transportation, domestic and gardening work, leisure time related activity and work related activity. For each domain, participants were required to recall the number of days in a week they had moderate, vigorous and walking activities over at least 10 min. For example, for the transportation domain, the questions included “During the last 7 days,for how many days did you walk for at least 10 min at a time to go from place to place?”.

The International Physical Activity Assessment Questionnaire (IPAQ)-long form [27] was used as a guide to generate each domain’s sub-score or total metabolic equivalent (MET)-minutes/week physical activity. IPAQ-long was used as a guide because it is a widely applied, reliable and valid measure of physical activity [28]. It is also an appropriate measure of physical activity in developing countries due to its evaluation across a spectrum of domains which requires energy expenditure [29] compared to surveys focusing only on leisure time activities [29]. One MET is measured as the energy spent per minute by sitting [8]. The MET values for walking, moderate and vigorous activities within each domain differ and are presented in Table 1 for reference. Within each domain the walking, moderate and vigorous activity MET-minutes/week was computed using the formula.

**Table 1:** MET values by type of activity in each domain

Domains	Type of activity	MET-values
Work	Walking	3.3
	Moderate	4.0
	Vigorous	8.0
Transportation	Walking	3.3
	Cycling	6.0
Domestic and Garden	Moderate yard chores	4.0
	Moderate inside chores	3.0
	Vigorous chores	5.5
Leisure-Time	Walking	3.3
	Moderate	4.0
	Vigorous	8.0

### Walking/moderate/vigorous MET-minutes/week = MET value * number of minute activity done * number of days activity done

The total walking, moderate and vigorous activity scores were then summed across the domains to produce the total physical activity score. The total physical activity score was then categorized into low, moderate and high groups based on the guidelines contained in IPAQ long form [27].

Physical activity was classified as high when respondents reported 7 days of any combination of walking, moderate-intensity or vigorous-intensity activities to achieve a minimum total physical activity of at least 3000 MET-minutes/week.

Physical activity was classified as moderate when respondents reported 5 or more days of any combination of walking, moderate-intensity or vigorous-intensity activities to achieve a minimum total physical activity of at least 600 MET-minutes/week. Respondents classified under the low physical activity or physically inactive category did not meet any of the above criteria.

Active participants are defined as those who meets the above physical activity criteria for moderate and high activity or one that met the recommendations of 30 min of moderate physical activity for 5 or more days in a week or 20 min of vigorous –intensity physical activity on at least 3 days every week or has reached a total physical activity level of more than 600 METs minute per week. Respondents that did not meet the physical activity requirements for the low category or has a total physical activity less than 600 METs-minutes in a week are physically inactive.

### Control variables

Past studies have shown that demographic, example age, gender, location and ethnicity and socioeconomic variables (income groups, employment status) have an influence on the prevalence of CVD risk factors. Therefore, it is important to control for these influences to obtain a rigorous understanding of the effects of physical activity on cumulative CVD risk factors.

### Statistical analysis

A chi-square test was initially conducted to identify the existence of a significant bivariate relationship between the covariates and outcome. Then, the influence of physical activity levels on cumulative CVD risk factors is captured by using the ordinal logistic regression approach for three different age groups of adults while controlling for potential confounders, such as the demographic and socioeconomic variables. This is because at different age groups the relative importance of the risk factors is expected to vary [17]. Respondents were grouped into the age groups of 35–49, 50–64 and 65 and above.

An ordinal logistic regression analysis (proportional odds model) was applied due to the ordinal nature of the dependent variable with the cumulative CVD risk factors ranging from 0 to 3 or more risks. Finally, the best fitted model was used. The crude or unadjusted odds ratio (cOR) and adjusted odds ratio (aOR) were computed using the regression analysis with a threshold value of 1.00 whereby cOR and aOR above 1.00 indicate high odds of high cumulative CVD risk factors and below 1.00 shows low odds of high cumulative CVD risk factors. The assumptions of ordinal logistic regression analysis which includes the test of parallel lines for all three of the models and model fit were tested at 10 % and 5 % significance levels respectively. Multicollinearity tests for each of the model were carried out using the variation inflation factor (VIF). VIF values did not exceed 10, and hence, indicate no collinearity issues among the predictor variables. All the analyses were performed using SPSS version 20.0 and with a significance level of 0.05 and 0.10.

## Results

Table 2 presents the prevalence and descriptive statistics of the variables included in the study. The average age of the respondents in the sample is 51.8 years with a standard deviation of 9.4 (mean ± SD = 51.8 ± 9.4). The response rate for each of the question were more than 65.7 %, with the highest for respondents’ location (100.0 %) and lowest for low physical activity (65.7 %). Since the variables in the study had missing values less than 50.0 %, all of them were included in the analysis [30]. The missing values for the variables in the study are missing completely at random and concurs to Little’s MCAR test results (*p*-value > 0.05). Also, the missing values did not exceed 40.0 % of the sample observation, which passed the Cronbach-Alpha statistics [31, 32]. The sample is marginally skewed towards females (56.5 %), rural residents (60.7 %) and Malays (77.2 %) but is close to the population in the location studied. The high rates of moderate (72.0 %) and vigorous (69.8 %) activity suggest that the respondents were generally physically active and only 31.9 % had low physical activity. Also, the results show high rates of hypercholesterolemia (71.1 %) and obesity (71.5 %) compared to other CVD risk factors. The bivariate associations show highly significant results (0.1 % significance level) between the demographic variables (age groups, location, gender and ethnicity) except for marital status and cumulative CVD risk factors (Table 3).

**Table 2:** Prevalence of demographic, socioeconomic status and cumulative CVD risk factors

Characteristics by	Prevalence, *n* (%)	Mean ± SD
*Demographic*		
Age		51.8 ± 9.4
35-49	3086 (42.4)	
50-64	3328 (45.8)	
65 and more	859 (11.8)	
Total	7273, MV = 3	
Location		
Urban	2859 (39.3)	
Rural	4417 (60.7)	
Total	7276, MV = 0	
Ethnicity		
Malay	5618 (77.2)	
Chinese	283 (3.9)	
Others	1373 (18.9)	
Total	7274, MV = 2	
Marital Status		
Married	6408 (90.2)	
Others	693 (9.8)	
Total	7101, MV = 175	
Gender		
Male	3165 (43.5)	
Female	4111 (56.5)	
Total	7276, MV = 0	
*Socioeconomic Status*		
Employment Status		
Employed	4062 (61.0)	
Unemployed	2595 (39.0)	
Total	6657, MV = 619	
Income Groups		
Less than RM 2000	4221 (69.5)	
RM 2000 – RM 5000	1330 (21.9)	
More than RM 5000	525 (8.6)	
Total	6076, MV = 1200	
*Physical Activity Level*		
Low	1522 (31.9)	
Otherwise	3256 (68.1)	
Total	4778, MV = 2498	
Moderate	3442 (72.0)	
Otherwise	1338 (28.0)	
Total	4780, MV = 2496	
High/Vigorous	3841 (69.8)	
Otherwise	1663 (30.0)	
Total	5504, MV = 1772	
*Outcome*		
Hypercholesterolemia		
Present	4504 (71.1)	
Not Present	1827 (28.9)	
Total	6331, MV = 945	
Diabetes		
Present	1137 (18.2)	
Not Present	5123 (81.8)	
Total	6260, MV =1016	
Tobacco Use		
Yes	952 (13.3)	
No	6209 (86.7)	
Total	5257, MV = 2019	
Obesity		
Present	5078 (71.5)	
Not Present	2021 (28.5)	
Total	7099, MV = 177	
Hypertension		
Present	2502 (34.4)	
Not Present	4188 (65.6)	
Total	6690, MV = 586	
Cumulative CVD risk factors		
Zero	628 (8.7)	
One	1913 (26.4)	
Two	2494 (34.4)	
Three or more	2218 (30.6)	
Total	7253, MV = 23	

Note: SD- Standard Deviation, MV- number of missing values

Total does not add up due to missing values

**Table 3:** Bivariate association between cumulative CVD risk factors and predictor variables

Variables	Cumulative cardiovascular disease (CVD) risk factors				χ^2^	*p*-value
	None, n (%)	One, n (%)	Two, n (%)	Three or more, n (%)		
Age Groups (in years)					353.39	<0.001****
35 to 49	378 (12.3)	982 (31.9)	1058 (34.4)	659 (21.4)		
50 to 64	186 (5.6)	751 (22.6)	1143 (34.5)	1236 (37.3)		
65 and more	51 (6.3)	157 (19.3)	284 (35.0)	321 (39.4)		
Location					31.88	<0.001****
Urban	188 (6.6)	742 (26.1)	1051 (37.0)	863 (30.3)		
Rural	440 (10.0)	1171 (26.6)	1443 (32.7)	1355 (30.7)		
Ethnicity					283.04	<0.001****
Malay	371 (6.6)	1355 (24.5)	1965 (35.1)	1909 (34.1)		
Chinese	29 (10.4)	86 (30.7)	88 (31.4)	77 (27.5)		
Others	227 (16.6)	472 (34.4)	440 (32.1)	232 (16.9)		
Marital Status						
Married	571 (8.9)	1676 (26.2)	2201 (34.4)	1960 (30.6)	5.423	>0.10
Others	45 (6.5)	176 (25.4)	246 (35.5)	226 (32.6)		
Gender						
Male	266 (8.4)	779 (24.7)	1036 (32.9)	1071 (34.0)	30.92	<0.001****
Female	362 (8.8)	1134 (27.7)	1458 (35.6)	1147 (28.0)		
Employment Status					5.865	>0.10
Employed	369 (9.1)	1086 (26.7)	1388 (34.2)	1218 (30.0)		
Unemployed	209 (8.1)	652 (25.1)	901 (34.7)	832 (32.1)		
Income Groups					7.612	>0.10
Less than RM 2,000	378 (9.0)	1123 (26.6)	1420 (33.7)	1297 (30.7)		
RM 2,000 – RM 5,000	96 (7.2)	344 (25.9)	475 (35.8)	412 (31.0)		
More than RM 5,000	40 (7.6)	139 (26.5)	195 (37.2)	150 (28.6)		
Physical Activity Level						
Low	338 (22.3)	419 (27.6)	488 (32.1)	274 (18.0)	0.906	>0.10
Otherwise	211 (6.5)	775 (23.9)	1138 (35.1)	1118 (34.5)		
Moderate	230 (6.7)	811 (23.7)	1209 (35.3)	1177 (34.3)	1.601	>0.10
Otherwise	327 (24.5)	578 (43.3)	324 (24.3)	107 (8.0)		
High/Vigorous	118 (3.1)	1459 (38.1)	640 (16.7)	1617 (42.2)	1.466	>0.10
Otherwise	344 (21.2)	415 (25.6)	543 (33.5)	321 (19.8)		

Note: Significance level at ****0.1 %, χ^2^ – Chi-square value, RM-Malaysian Ringgit

Table 4 shows the adjusted effects of physical activity levels on the incidence of cumulative CVD risk factors among the three different age groups, 35–49 (Model I), 50–64 (Model II), and 65 and above (Model III). The results show low odds of having high cumulative CVD risk factors among those in the age group of 35–49 years and who perform moderate compared to reference category; low levels of physical activity (aOR_moderate_:0.12; 95 % CI: 0.02 - 0.53) and is significant at 1 % significance level. Also, those who are between 50 to 64 years of age and living in urban areas are less likely to develop high cumulative CVD risk factors (aOR: 0.82; 95 % CI: 0.64-1.02) compared to others in the sample. In other words, the odds of having high cumulative CVD risk factors of respondents living in rural areas is 1.23 (exponential (0.208): 1.23) compared to urban dwellers. However, physical activity showed no statistically significant effect on the prevalence of cumulative CVD risk factors among people in the 50–64 age group compared to low physical activity. The elderly group of people aged 65 years and above who perform vigorous physical activity showed a low prevalence of cumulative CVD risk factors compared to low physical activity. The odds of Malays in this age group (aOR: 2.51; 95 % CI: 1.86-7.40) attaining high cumulative CVD risk factors is thrice as likely as those in other ethnic groups. Furthermore, the assumptions of ordinal logistic regression is met whereby the test of parallel lines for all three of the models were insignificant and the model fit was significant at 10 % and 5 %.

**Table 4:** Fitted estimates of predictor variables by means of ordinal logistic regression analysis

Characteristic	β	aOR, (95 % CI)	β	aOR (95 % CI)	β	aOR (95 % CI)
Ethnicity						
Malay	−0.228	0.80 (0.18, 3.50)	0.466	1.59 (0.91, 2.79)	0.923**	2.51** (1.86,7.40)
Chinese	0.068	1.07 (0.02, 52.35)	−0.065	0.92 (0.45, 1.93)	0.871	2.39 (0.54,10.61)
Others	(ref.)	1.00	1.00	1.00	1.00	1.00
Location						
Urban	-	-	−0.208*	0.82* (0.64, 1.02)	-	-
Rural	-	-	(ref.)	1.00	-	-
Marital Status						
Married	1.14	3.13 (0.04, 2.73)	−0.058	0.94 (0.71, 1.59)	0.11	1.12 (0.64, 1.96)
Others	(ref.)	(ref.)	1.00	(ref.)	(ref.)	(ref.)
Gender						
Male	-	-	0.148	1.16 (0.92, 1.46)	-	-
Female	-	-	(ref.)	(ref.)	-	-
Income Groups						
Less than RM 2,000	−0.674	0.51 (0.14, 1.81)	-	-	-	-
RM2,000 – RM 5,000	−0.916	0.40 (0.12, 1.37)	-	-	-	-
More than RM 5,000	(ref.)	(ref.)	-	-	-	-
Employment Status						
Employed	0.466	1.59 (0.66, 3.86)	-	-	-	-
Unemployed	(ref.)	(ref.)	-	-	-	-
Physical Activity Level						
Moderate	−2.141***	0.12*** (0.02, 0.53)	0.191	1.62 (0.74, 1.98)	1.10	3.00 (1.09, 8.28)
Vigorous	0.998*	2.71* (0.83, 8.87)	−0.138	0.98 (0.58, 1.32)	−0.547**	0.58** (0.24, 0.78)

Note: Significance level at * 0.10, ** 0.05, ***0.01, ref. – reference category, RM- Malaysian Ringgit, aOR-adjusted odds ratio, β – coefficients, CI- Confidence interval

Model I represents the regression for age group 35–49, Model II for age group 50–64 & Model III age group 65 and above. Model I includes all the predictor variables except location and gender. Model II includes all the predictor variables except employment status & income groups. Model III includes all predictor variables except gender, employment status, income groups and location

## Discussion

The results of our study concurs with the findings of previous studies done in United Kingdom [16], United States [13], Norway [17] and Finland [14], which showed that physical activity reduces the likelihood of CVD risk factors. However, our results differ from other studies in Europe [20] and Spain [21] that showed physical activity is positively associated with CVD risk factors. The latter could be a consequence of respondents engaged in too strenuous physical activity while being afflicted with CVD risk factors, which suggests the need to establish threshold levels of physical activity among those suffering from CVD risk factors.

The findings of our study also show that those with moderate physical activity in the age group of 35–49 had low probability of having three or more CVD risk factors. This positive effect questions the unusual results of a study conducted in Chennai, India, which showed high incidence of CVD risk factors (diabetes, obesity, abdominal obesity, hypertension, dyslipidaemia) among those that carried out light physical activity [15]. People in this age group generally have a working class background and are likely to experience moderate to low energy expenditure and low calorie food, which may explain the inverse effects of moderate physical activity on cumulative CVD risk factors.

Our results show no statistical significance between moderate and high levels of physical activity, and cumulative CVD risk factors among respondents in the age group of 50–64 compared to the low physical activity. The lack of statistical significance in this age group could be a consequence of respondents nearing retirement who could be faced with the stress of pondering over their future. Hence, stress should be included in future studies to see if it has a bearing on the relationship between physical activity and cumulative CVD risk factors.

As the aging process takes place, people are incapable of participating in activities that require high energy intake, which is apparent in almost all the regions documented by the World Health Organization (WHO) [9]. Although this trend is appearing in many nations around the world, it does not hold for elderly people above the age of 60 years in South-East Asia region [9]. Our results substantiate this trend among those aged 65 and above, which shows an inverse association between vigorous physical activity, and high cumulative CVD risk factors compared to low physical activity. Our results also concur with the findings of another study in Norway, which showed low hypertension and diabetes rates among those above 65 years as physical activity increased from low to high levels [17].

Also, our results show that rural respondents aged 50 to 64 years and Malays in the age group of 65 and above years are more likely to develop high cumulative CVD risk factors than others. However, this contradicts the findings of a study that showed that urban people in middle income countries are more likely to attain risk factors associated with CVD [25]. That rural people in the 50–64 age group are prone to having cumulative CVD risk factors may be a consequence of a number of them still living in land tenure schemes so that their retirement may have left them vulnerable as their children have largely migrated to urban locations [33]. Nevertheless, our results are consistent with results found in the high income countries using the INTERHEART risk score [25]. Also, past studies show that the prevalence of CVD risk factors is generally higher among Malays than the Chinese, which may explain the peculiar results found in Malaysia [34, 35].

## Conclusion

The results of this paper show that physical activity lowers cumulative CVD risk factors among respondents in the age groups 35–49 and 65 and above, which supports the case for introducing intervention programmes, such as provision of facilities and built environment that allures physical activity in community parks, workplaces, schools and colleges to shape a healthy community. However, the results were not significant among respondents in the age group 50–64, which raises the question of whether there are threshold levels for physical activity and if other influences, such as stress levels and food consumption by age group and exposure to environmental pollution may require equally important focus. Future studies should investigate these influences to focus policy better on reducing the prevalence of cumulative CVD risk factors. Also, given similar CVD risk factor trends among Southeast Asian economies it may be advisable to carry out region-wide studies in future [9]. Such efforts may also lead to regional collaboration among these nations to combat the rising prevalence of CVD risk factors in the region.

### Strengths & limitations

The main strengths of the present study are a large sample size and available anthropometric and clinical data that was measured during health screening. Although the sample is not a national random sample, it includes different geographical areas, demographic and socioeconomic groups and states in Malaysia which enabled a regional analysis. This study has some limitations that should be overcomed by future studies. The causal effect of physical activity on CVD risk factors or vice versa was not accounted for owing to the cross-sectional data used. The findings of this study can be strengthened by a longitudinal assessment of changes in the impact, if any, of physical activity on CVD risk factors. Another limitation of the study is that the sample used a convenience sampling design that may be exposed to selection bias. Also, the outcome variable, cumulative CVD risk factors were assigned equal weights assuming that the risk factors were equally likely to have an impact on the development of CVD. However, some CVD risk factors have greater degree of severity in the development of CVD compared to others [36].

## Abbreviations

CVD: Cardiovascular disease
NCD: Non-communicable disease
CHD: Coronary heart disease
BMI: Body mass index
MANS: Malaysian Adult Nutrition Survey
HDL: High density lipoprotein
LDL: Low density lipoprotein
REDISCOVER: Responding to Increasing Cardiovascular Disease Prevalence Study
UiTM: Universiti Teknologi MARA
TC: Total cholesterol
IPAQ: International Physical Activity Assessment Questionnaire
MET: Metabolic equivalent task
VIF: Variation inflation factor
SD: Standard Deviation
RM: Malaysian Ringgit
aOR: Adjusted odds ratio
cOR: Crude odds ratio
CI: Confidence interval
ref.: Reference category
WHO: World Health Organization
